# Response letter to “Latent class analysis of 216 patients with adult-onset Still’s disease” by Sugiyama et al.

**DOI:** 10.1186/s13075-022-02984-7

**Published:** 2022-12-28

**Authors:** Marion Delplanque, Arsène Mekinian, Sophie Georgin-Lavialle

**Affiliations:** 1 Sorbonne Université, Service Médecine Interne, Centre de référence des maladies autoinflammatoires et des amyloses (CEREMAIA), APHP, Hôpital Tenon, Paris, France; 2 Sorbonne Université, Service de Médecine Interne, APHP, Hôpital Saint Antoine, Paris, France

**Keywords:** Adult-onset Still’s disease, Myelodysplastic syndrome, VEXAS

## Abstract

Sugiyama et al. recently described in “Latent class analysis of 216 patients with adult-onset Still’s disease,” baseline characteristics, laboratory tests, treatment, relapse, and death of adult-onset Still’s disease (AOSD) patients from a Japanese hospital. They identified two subgroups: Class 1 (*n*=155) with a younger age and typical symptoms of AOSD and Class 2 (*n*=61) with older patients and fewer typical symptoms of AOSD. In 2022, VEXAS (vacuoles, E1 enzyme, X-linked, autoinflammatory, somatic) syndrome, an established X-linked disease associated with a somatic mutation in *UBA1*, is considered as a differential diagnosis for AOSD particularly in elderly. These patients from Class 2 could benefit from more explorations for mild myelodysplasia and VEXAS.

Dear Editor,

With great interest, we read the recent article entitled “Latent class analysis of 216 patients with adult-onset Still’s disease,” published online in your journal. In this paper, Sugiyama et al. performed a retrospective latent class analysis about baseline characteristics, laboratory tests, treatment, relapse, and death of 216 adult-onset Still’s disease (AOSD) patients in nine Japanese hospitals. They were able to identify two subgroups: Class 1 (*n*=155) with a younger age and typical symptoms of AOSD and Class 2 (*n*=61) with older patients with a median age of 66.8 vs 41.5 years old in Class 1 and fewer typical symptoms of AOSD.

Recently, Beck et al. [1] described a newly established X-linked disease associated with a somatic mutation in *UBA1*, a gene encoding ubiquitin-like modifier activating enzyme 1 and named VEXAS for vacuoles, E1 enzyme, X-linked, autoinflammatory, somatic syndrome. It is characterized by fever, skin lesions, lung infiltrates, but also arthralgia, lymph node enlargement, and macrocytic anemia, mostly among elderly men [2]. In 2022, VEXAS is considered as a differential diagnosis for AOSD particularly in elderly [3] as illustrated in a recent retrospective Dutch case series of 12 patients with adult-onset auto-inflammation caused by somatic mutations in *UBA1*, two were initially suspected with AOSD [4]. Interestingly, Class 2 patients of Sugiyama et al.’s work seem to present deeper anemia (10.6 vs 11.2g/dl) according to supplementary table 1, it could be inflammatory anemia but also partly macrocytic anemia so it would be useful to know the mean corpuscular volume of each group. In the same way, patients of Class 2 experienced less typical skin rash but did they have other cutaneous features or other atypical symptoms?

Therefore, we think that it would be very interesting to have patients from Class 2 group screened for myeloid neoplasm at least and for *UBA1* mutation in Sanger technique in order to know if this identified group could be patients with autoinflammation linked to *UBA1* mutation or another somatic mutation not identified yet.

Patients in the Class 2 group may not all be VEXAS syndrome, considering the female predominance, but their age and, for example, the presence of two cases of interstitial pneumonia in a small group of 61 should encourage for more explorations including VEXAS and mild myelodysplasia [5, 6].

## Data Availability

Data sharing is not applicable to this article as no datasets were generated or analyzed during the current study.

## Abbreviations

AOSD: Adult-onset Still’s disease
UBA1: Ubiquitin-like modifier activating enzyme 1
VEXAS: Vacuoles, E1 enzyme, X-linked, autoinflammatory, somatic syndrome
